# Human Development X: Explanation of Macroevolution — Top-Down Evolution Materializes Consciousness. The Origin of Metamorphosis

**DOI:** 10.1100/tsw.2006.270

**Published:** 2006-12-15

**Authors:** Tyge Dahl Hermansen, Søren Ventegodt, Joav Merrick

**Affiliations:** ^1^ Quality of Life Research Center, Teglgå rdstræde 4-8, DK-1452 Copenhagen K, Denmark; ^2^ Research Clinic for Holistic Medicine, Copenhagen, Denmark; ^3^ Nordic School of Holistic Medicine, Copenhagen, Denmark; ^4^ Scandinavian Foundation for Holistic Medicine, Sandvika, Norway; ^5^ Zusman Child Development Center Soroka University Medical Center Ben Gurion University of the Negev, Beer-Sheva, Israel; ^6^ National Institute of Child Health and Human Development, Jerusalem, Israel; ^7^ Office of the Medical Director Division for Mental Retardation Ministry of Social Affairs, Jerusalem, Israel

**Keywords:** holistic biology, theoretical biology, evolution, Denmark

## Abstract

In this paper, we first give a short discussion of the macroevolution viewing life as information-directed, complex, dynamic systems. On this basis, we give our explanation of the origin of life and discuss the top-down evolution of molecules, proteins, and macroevolution. We discuss these subjects according to our new holistic biological paradigm. In view of this, we discuss the macroevolution of the organism, the species, the biosphere, and human society. After this, we discuss the shift in evolution from natural selection to a new proposed process of nature called the “metamorphous top-down” evolution. We discuss the capability of the evolutionary shift to govern some of the processes that lead to the formation of new species. We discuss the mechanisms we think are behind this proposed shift in evolution and conclude that this event is able to explain the huge biological diversity of nature in combination with evolutionary natural selection. We also discuss this event of nature as an isolated, but integrated, part of the universe. We propose the most important genetic and biochemical process that we think is behind the evolutionary shift as a complicated symbiosis of mechanisms leading to metamorphosis in all biological individuals, from bacteria to humans. The energetic superorbital that manifests the consciousness governs all these processes through quantum chemical activity. This is the key to evolutionary shift through the consciousness, and we propose to call this process “adult human metamorphosis”.

## INTRODUCTION

In this paper, we will discuss the capability of nature to create the huge amount of biological diversity that has been formed through evolution. As this is a theoretical discussion, we only guess and suggest how nature governs this incredible ability to create new species. Therefore, the written text may here and there give the expression that we think this is the definite truth, but our purpose is only to suggest a new explanation of these functions of nature, because we have not found that the existing knowledge is able to explain these things in detail. Therefore, we have developed a new way to explain events of nature that have not been explained like this before. We believe it is a step towards the truth and believe this explanation can work until something better has been developed. It is not our intention to summarize the modern evolutionary biology's theories of the relation between form and function; this has been done elsewhere[1,2,3,4].

Awareness, intention, and will are the driving forces behind morphogenesis. When it comes to the description of man, it is evident that the biological information that is buried deeply in the lower levels of the biological system manifests itself by means of organization of the complex dynamics of the organism as a whole. This organization corresponds to a certain intention and creates a specific interaction between the wholeness and a certain item with a corresponding pattern[5]. If the pattern of the wholeness corresponds with organs and organ systems, willpower as the organizing force is directed towards these and the organization of the organism is enforced. This is the morphogenesis interpreted through the intention. The interpretation is particularly relevant because it implies that the organization of the organism is functioning because of an intention to do so[5]. In principle, there is no difference between an intention directed towards outer or inner organization. In this paper, we discuss macroevolution on earth from the evolution of an organism to the evolution of human society.

The evolutionary activity of nature usually is described as happening through natural selection[6]. We think that such an evolutionary event is not capable of describing the great diversity of biological organisms developed through evolution of life and existing on the earth today. Therefore, there has been a pressing need for a new evolutionary theory from evolutionary scientists, to be able to explain the evolution of diversity in greater detail. A new evolutionary hypothesis capable of satisfying these needs is a new "fly-wing theory" (paper under preparation for publication). Following this, the fly developed its wings through metamorphosis in a process called the "metamorphous top-down" evolution.

The capability of nature to create new species and thereby increase biodiversity was a main problem of evolutionary science until Charles Darwin (1809–1882) published his book *The Origin of Species* in 1859[6]. He gave scientists a tool that was powerful and partially able to explain the huge biodiversity that had existed on earth. But to fulfill the needs for nature to create this huge biodiversity, we think that something else has to interact with the Darwinian "natural selection". Therefore, we have proposed the "metamorphous top-down" evolution as a tool for nature to create biological diversity in a tight interaction with the natural selection proposed by Darwin (in the fly-wing theory in preparation for publication).

Charles Darwin based his evolutionary theory of "natural selection" on a "bottom-up" development, where the evolution went from varieties to taxa, based on the idea that a variety that represents the trait that makes an individual most fit for survival will eliminate the others through natural selection[6] (already Darwin knew that insects have sperm from 20 males to choose between, and all individuals choose, especially the females). On the other hand, the principle of "metamorphous top-down" evolution is based on the capability of an individual and consciousness to go through a process of metamorphosis that develops a specific trait necessary for the individual to stay fit and survive in its habitat or microhabitat, and thereby give a great contribution to the diversity of living organisms existing on the earth.

Below, we will first discuss macroevolution as a consequence of evolution and, afterwards, some of the mechanisms behind the great amount of diversity developed through evolution. We discuss the capability of "metamorphous top-down" evolution to force the creation of new species, and we will do this by looking at it as a local event that is an integrated part of the whole universe, but acts as its own "tiny" (read local) universe, as a small part of the whole. This is not a religious explanation of how God interacted with nature and created the species, but as a tool for scientists to understand some of the mechanisms of nature that lead to the creation of new species, and to try to see this "tiny" event in a greater perspective by looking at it in a fractal manner compared to the universe as a whole. By doing this, we will try to make readers understand that this is only a little isolated event of the universe, but it demands a huge amount of coordination and power to be able to happen.

## MACROEVOLUTION OF THE BIOSPHERE AND ITS BIOLOGICAL LIFE

Concerning the origin of life, it is unlikely that the morphological information of the organism is situated in the DNA base sequences because of (1) the C-value paradox, e.g.[7]; (2) normal self-organization of proteins is unable to explain the general morphological features[8]; and (3) form can apparently evolve without corresponding molecular evolution[4,9]. Supposing that the information giving form lies in the fine structure of energy, the amount of information is not a problem and, likewise, the information transport becomes understandable. We believe that the origin of life can be given a reasonable explanation. According to our hypothesis, the fine structure of energy has an inherent ability to drive the self-organization of all levels, from the smallest units to prokaryotes (bacteria and the like), unicellular to multicellular eukaryotes. We believe that there is a creative principle in nature in which evolutionary selection works. We do not think this is caused by mutations in DNA because proteins do not give rise to form in itself[9]. Therefore, we believe it makes perfect sense if the fine structure of energy works in a creative way and organizes evolution. The evolutionary leaps that hitherto have lacked an explanation, e.g., the evolution from unicellular to multicellular organisms, can also be explained through our hypothesis, since the creative principle works through creation of still higher organization levels. The more-or-less constant mutation rates of DNA are called microevolution in contrast to nonmolecular evolution, which is usually called macroevolution. In this paper, we discuss macroevolution in view of our new holistic biological paradigm.

*Top-down evolution of molecules:* The evolution of function in the first era of life on earth, presumably lasting the first couple of billion years, was strongly dependent on molecular evolution greatly assisted by the ongoing evolution of shape and function. Molecular evolution was, we believe, also guided by high-level organizational forces. If modern day bacteria mirror the early bacteria, by recombining DNA[9] they were able to produce new proteins and if the informational system was as smart as we envision it, it could even tell immediately if a protein would be useful for a certain intended task. This is very much the way bacteria gain resistance towards antibiotics today. The recombination part of molecular evolution might be a random process, as we today do not believe that the cell controls the small molecules like DNA fragments, which are subject to strong Brown's stochastic movements. We also believe that molecular evolution was strongly influenced by natural selection.

*Macroevolution of the organism:* The macroevolution of the organism can be divided into the following parts: evolution of the inner organs[10], and evolution of the brain and the limbic apparatus[11]. The evolution of the structure of the inner organs was narrowly tied to their function. The function of an organ can be thought of as a result of its structure, however, the function has to act on the structure through the system's evaluation and later modification of the structure. Structure may be an evolutionary product of function instead of the other way around. This demands that the biological system has a double representation of its structure and its function. This corepresentation may be the foundation of the evolution of intension in the biological system.

*The macroevolution of the species and the biosphere:* The macroevolution of the limbic apparatus and the brain as we recognize it from the vertebrates that all have the same muscles, bones, brain building plane, etc.[4], but different macroscopic shape of all its parts, counts in a very high degree on adaptation after the surroundings and specific circumstances[6]. This can be understood by looking at all individuals within a species through specific, information-transforming interactions in its biosphere. Such interactions make it possible for each individual to interact with the others within the same species, and the same counts for other living individuals in their milieu.

Now, we will take a little longer look at the interaction that all organisms in a biosphere may have with each other. All living beings seem to have the ability to represent all the other organisms as well as higher levels of organization in their ecological niche. The function of will and attention is understood as the capability to transform the representation of one's own organization to other life and represent other life in oneself, that recognizes biological systems in general[5]. This counts also for plants, sponges, and single-celled animals. This was exactly the original premise for the formation of multicellular organisms. All ecosystems are developed through a long time of evolution, where all life forms arranged themselves in accordance to each other and may have influenced each other's evolution through the capability to force a representation. An example of governing the development of life has appeared through our house animals. An example is the dog. Dogs have been bred for almost all purposes: gundogs, watchdogs, lapdogs, sheepdogs, etc.

Normally it is accepted that the development happens through natural selection. The consequence of the description of life as a complex dynamic is that the development in the same degree happens through arrangement to the other life in the biosphere. Exactly; to a very high degree, the human being has disengaged itself from this arrangement through the use of its reason and through technical development. Conforming also counts under the direct use of the will in the highest degree to the emotional interpretation of reality.

Life is developed through common arrangement, through an ongoing development of the capability to use the physical circumstances. Behind the evolution is a driving, organizing power as is in the structure of the energy itself (compare with the spiral fractal).

*Macroevolution of the human society:* Information-transmitting interdependencies between man as an individual and the higher organizational levels of the human communities are a necessity for the social function of the individual. The functionality of the family is completely dependent on the ability of the participants to adapt to a community. Human society should be viewed as a biological structure in itself, which is under evolutionary development. The process of democratization is a beautiful expression of the evolution of mankind towards integration into a greater society. The human society is a very complex biological structure, much more complex than an anthill, however, in principle, there is no difference. When mankind learns to respect the whole, it will at last be able to function in the world in a constructive way.

## What Drive in Evolution Created the Diversity on Earth?

What has been driving evolutionary development in this direction? What forces of nature are capable of creating a world so diverse that a biosphere can cover a huge symbiotic ecosystem as the main ecosystem of earth? We think that powers like natural selection and "metamorphous top-down" evolution interact with each other to be able to create a biodiversity necessary for the ecosystem of the biosphere to survive. Below we will describe what we think makes the evolutionary drive for biodiversity. We will discuss the mechanism in nature that drives the evolutionary shift from natural selection to "metamorphous top-down" evolution. This will be discussed in detail from the advanced biochemical and genetic processes involved in this shift to the perspective of an isolated event of nature compared to the great wholeness of the universe.

## The Force that Drives the Shift in Evolution is Governed by Quantum Energy

The shift in evolution, from our point of view, between Darwin's natural selection and the "metamorphous top-down" evolution is governed by quantum chemical activity directed from the superorbital[12] of nature combined with the superorbitals of the individual organisms involved in the evolutionary event, through their consciousness. The individual's consciousness is driven by the need for survival in a specific habitat or microhabitat. The natural consciousness is driven by the need for nature to keep the biological diversity on the earth (GAIA) as enormous as possible. We imagine that the superorbitals of the involved parts gain a kind of resonance with each other and, through quantum mechanical activity, this resonance activates the energy necessary to satisfy the needs of the individuals for survival in their habitat and the needs of nature to gain a more complex diversity. Such activity forms a gathered superorbital for the involved parts that drives the development against formation of new species. In this way, new species can be formed that will be fit to survive in all kinds of milieus and all habitats and microhabitats. Such a mechanism is very practical for nature, because the quality of nature to keep species alive is very dependent on an extremely complex diversity of living organisms on the earth[13]. This means that the shift in development is a kind of feedback mechanism that is turned on to satisfy the needs of earth for more diversity, to gain survival as a huge symbiotic ecosystem, and to satisfy the individual organism's needs to adjust for survival when natural selection fails to satisfy these needs. This means that nature has created the "metamorphous top-down" evolution as a mechanism that is able to interact with natural selection when it is necessary for the single individual to adjust itself because natural selection was not able to fulfill these needs, and the individual has got the capability to drive this event by its consciousness. Likewise, by its consciousness, nature itself is capable of driving this force when natural selection fails to create a diversity of living organisms necessary for survival of the huge symbiotic ecosystem that is manifested in the biosphere of the earth.

We think that such powers are working all over in nature because it is the way of nature to guard itself against destruction. The main power of nature is interaction between energies that shape a superorbital for each single component in nature. Combined, they will shape a huge superorbital gathering all superorbital energies in universal consciousness, capable of organizing the whole universe (nature) in one huge symbiotic "organism". This "symbiosis" represents the highest fractal level and could, in religious terms, cover the term God, but in traditional scientific thinking, it covers the capability of the universe to coordinate all functions within the universe in each tiny little detail. It is the same kind of power that drives the shift in evolution, but on a lower fractal level. The gathered superorbital of nature and the individuals involved in such an evolutionary event make their own "tiny" universe, governed by the superorbital that delivers the quantum mechanical energy for driving the shift in evolution.

If we look at the evolutionary shift from "natural selection" to "metamorphous top-down" evolution as an isolated event, then it is obvious to imagine that this is a very complicated and sensitive process in itself that involves many complicated processes that have to cooperate in a very finely timed interaction. These interactions involve biochemical activity and master gene control of the single individual organisms involved in the evolutionary event in order to direct the process of metamorphosis in a given direction. In this way, nature gets its need for more complex biodiversity satisfied and the individual organisms get their need for developing a peculiar trait necessary for survival in a specific habitat or microhabitat.

Below we will describe some of the processes that we think are involved in "metamorphous top-down" evolution, governed in following sequences: chemical bombing raid → biochemical reactions → genetic control → morphogenesis → manifested mirror image → genetic regime control → parallel metamorphosis of several individuals leading to a new species. We think these processes are tightly regulated by the orbital mastering the processes, and we will try to describe our thoughts concerning the interactions between quantum mechanical activity of the superorbital, the biochemical and regulatory processes happening in the single biological organism through metamorphosis.

## Biochemical and Genetic Interactions

When nature shifts from evolutionary natural selection to "metamorphous top-down" evolution, an extremely large and complicated apparatus, involving a huge amount of strict and coordinated processes of genetic and biochemical origin, has to happen for these powerful processes to function as the "symbiotic" wholeness they are. Below we will try to give our explanation for how this coordination can take place by going through a number of genetic and biochemical activities that we think are important candidates behind the shift in evolution involved in increasing the biodiversity of the earth.

### *Chemical Bombing Raid*

The first chemical signal for forcing the event of "metamorphous top-down" evolution is a chemical bombing raid. This is governed by the quantum chemical energy from the superorbital. This initiates the activity of genes involved in production of pheromones and other airborne ligands necessary to initiate a biochemical reaction in individuals having the need for the same peculiar trait for survival as the donor organism. This can be individuals in the same habitat or, because the ligands are airborne, individuals from other populations around the world in need of the same trait in order to survive in their habitats. Such ligands trigger the biochemical activity that leads to genetic control by getting absorbed through the mucous membranes of the nose or through membranes of other olfactory organs. Through the body liquids, they are transported to the surface of the body cells where they interact with protein receptors that change conformation and are thereby strictly controlled from the consciousness arisen from the superorbital[12]. Such activity initiates multiple processes and interactions in the cell.

## Biochemical Reactions

The conformation change of the cell surface receptors[9,12] is the first biochemical reaction in the sequence of events leading to metamorphosis. All processes initiated in the cell by this action are of biochemical and genetic character. Such actions give the cell the possibility to adjust itself to processes leading to organ and tissue production, and in the end, the capability of forming a complete adult individual organism with its own consciousness. Changing of protein conformations of the cell surface informs the superorbital (consciousness)[12] about initiation of metamorphosis[14]. Thereby, the superorbital adjusts its energetic activity to coordinate a long row of biochemical activity, leading to control of master genes involved in the morphogenesis. This includes a row of feedback mechanisms that involve genetic activity for production of master regulatory proteins that regulate the expression of the metamorphous master genes. It also leads to control of the processes of cell cycle control and control of cell proliferation. These processes also include huge amounts of biochemical activity, and control of master genes and master regulatory proteins. From this it is easy to imagine the huge "symbiosis" of processes and mechanisms lying behind the united process that initiates the "metamorphous top-down" evolution.

## Control of Master Genes and Master Regulatory Proteins

The metamorphous process itself is governed through activity of master genes and master regulatory proteins. A lot of master genes have been identified that are involved in the process of metamorphosis through evolution (a summary of some of the most important of these are in preparation for publication). Such genes produce proteins that are involved in the regulation of expression of all other genes of the single individual's genome, called master regulatory proteins[9]. This means that the superorbital, by consciousness, through strict and precisely energetic control of the quantum chemical energies needed for expression of genes necessary for all developmental processes of a forming body to process, is able to control each tiny little biochemical process needed for "metamorphous top-down" evolution. This includes production of hormones and other ligands needed for configuration changes of proteins, membrane proteins involved in cell to cell adhesion and transport of ions through the cell membrane, ATPase and ATPsynthetase involved in production and degradation of ATP (the main energy resource of the cell)[9,15], but also production of proteins involved in regulation of mitochondria regulatory proteins and thereby involved in the main production of quantum chemical ATP energy of the cell[15], that again is under strict control from the superorbital. And this amazing symbiosis of processes controlled from the superorbital gives only a humble overview of a little corner of the biochemical processes of nature, and may be considered as much more complex and complicated in reality, because scientists have only been able to map a bit of the great whole of processes and mechanisms involved.

## Morphogenesis

The control of master genes and master regulatory proteins, as mentioned above, leads to the metamorphous processes necessary for nature and individual organisms to satisfy their needs for fitness and survival. These processes make the gathered superorbital of the individual organisms and nature (their joint consciousness) capable of, through influence of the quantum mechanical orbital energy needed for positional information on the different fractal levels, organizing its own evolution of such traits through developmental enforcement of the traits necessary for survival in a specific habitat or microhabitat. Concerning nature, such processes make it capable of, through the gathered superorbital, forcing the single individuals involved in the processes to go through the "metamorphous top-down" evolution for satisfying the natural need to multiply the diversity of species necessary for keeping the huge gathered ecosystem of the earth fit and stable[13].

## *Manifested Mirror Image*

Following the hypothesis of "metamorphous top-down" evolution, this process is manifested as a mirror image that influences the whole species genetic regime, and through the airborne diffusion of chemicals during the chemical bombing raid, such a mirror image can be manifested in isolated habitats that need the same peculiar traits for survival and thereby give the capability of developing different species with, for example, wings, such as the species of the class Diptera that includes the fly, gnat, and mosquito. Such functions are governed through the diffusion of pheromones carried by the wind and other airborne proteins secreted by an individual through chemical bombing raids as mentioned above and through changes in behavioral patterns that again implement a huge amount of biochemical reactions governed from the superorbital. Such processes implement the same kind of biochemical action governed from the superorbital as mentioned earlier, but in the connection with "manifested mirror image", the behavioral patterns that are implemented between the individuals within a habitat are of major importance. It is those that make the difference between the manifested mirror developments in the different populations. Thereby, they force the different populations against different development and, by this, force the development of an even more complex diversity of species. This means that in each isolated habitat, through the internal chemical bombing raid and behavioral activity in combination, the development goes through the evolution of a new species (see Fig. 1), but in different isolated habitats such development leads to different species (not identical but "of the same kind"), e.g., gnat, mosquito, and the fly of Diptera. This means that different behavioral patterns involved in the implementation of a manifested mirror image are the reason why some isolated populations develops to gnats, others to flies, and even others to mosquitoes. Such developmental differences are caused by very small differences in the positional information of the different fractal levels[16,17,18], leading to small changes in the evolution of a population through the ontogenesis of the adult individual, and are under tight control from the superorbital gathered between nature and the involved individuals. This means that these use their gathered consciousness to gain their needs for survival.

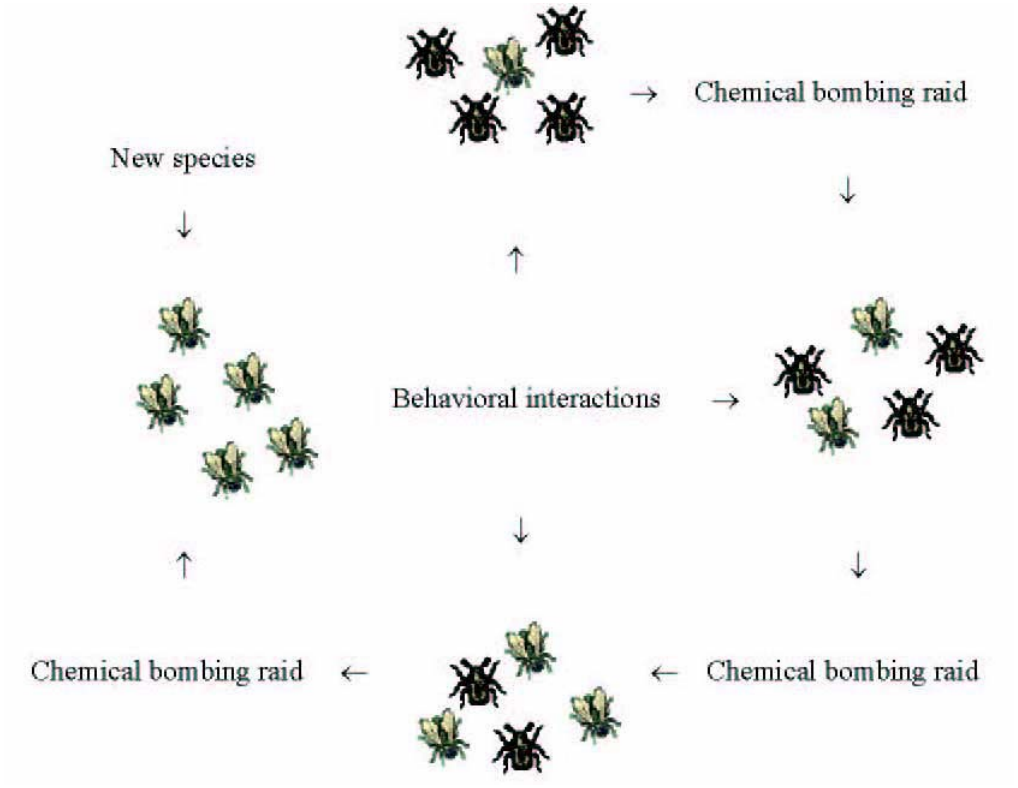

**FIGURE 1.** In a population, when there is a breeding ground for the development of a specific trait, e.g., the development of wings, this will gain behavioral patterns that favor the development of wings in the population through activation of biochemical reactions. At the same time, chemical substances are transferred between the individuals, also initiating biochemical reactions. Such biochemical reactions ensure the control over the genetic regime, so more and more individuals develop the wings. The more individuals that have the wings, the greater evolutionary pressure there is in the population in favor for wing development. In the end, this will involve the creation of a new species.

## *Genetic Regime Control*

The manifested mirror image leads to "genetic regime control". This means that the gathered superorbital gets control over the genes of all biological organisms involved in the development of a specific species in a way so it is capable to coordinate the genetic activity towards developing "morphologically identical" individuals within the new species. Such control is governed by the superorbital[12] through parallel organization of the distribution of positional information at the different fractal levels[16]. In this way, the gathered superorbital is able to guide the development of a new species. Thereby, both nature and the individual organisms governed from the superorbital have their needs covered. Nature has expanded the diversity of species and the individuals get their need for survival satisfied.

## The Process of Adult Human Metamorphosis

"Metamorphous top-down" evolution is driven by the gathered superorbital. The superorbital consists of all energies involved in all processes involved in the evolutionary shift. This is also our definition of consciousness[12]. Therefore, the shift in evolution is governed by the consciousness. By this interpretation, the consciousness is involved in the development of a huge amount of species and peculiar traits of individual biological organisms through evolution from bacteria to humans. We think that such an evolutionary mechanism as the "metamorphous top-down" evolution has been interacting with the mechanisms of natural selection through evolution, and these two mechanisms together are able to explain the huge diversity evolved through evolution. This process of “metamorphous top-down” evolution has also been able to force the development of a more realistic consciousness with the development of more complex organisms. We also have a kind of feedback function. Consciousness forces the “metamorphous top-down” evolution to happen, and the “metamorphous top-down” evolution evolves a more complex consciousness, leading to human consciousness as, until now, a temporary end of the evolution. What comes next? We do not think evolution has stopped yet. As a very “advanced” result of the “metamorphous top-down” evolution, humans have evolved the capability to go through an adult human metamorphosis that leads to recovery from illnesses and distortion, in order to gain fitness and survival. The adult human metamorphosis is a delicate kind of “metamorphous top-down” evolution and is probably capable of leading the development of humans to new heights of evolution.

## DISCUSSION

This paper discusses the mechanisms behind the evolutionary shift between “natural selection” and “metamorphous top-dawn” evolution. We see the processes involved in this evolutionary event as a function of consciousness. The consciousness drives evolution to make its shift by a tight and strict regulation of quantum chemical energies that are governed by and are a part of the superorbital. Nature uses the fractal structural levels to govern the distribution of positional information in a way that leads to the creation of new species. In this way, the shift in evolution is able to satisfy both the earth’s needs for the creation of new species, and the single individual’s needs for fitness and survival. In this way, nature is able to continue and maintain its biological characteristic as a huge symbiotic ecosystem covering the whole biosphere of the earth, through consciousness.

We imagine the main power of nature (read universe) as one complete symbiosis of energies gathered in one huge superorbital, and isolated events in nature as the shift in evolution, as a local tiny “universe” acting in itself, but as a part of the great wholeness of nature. Such local “universes” are the building blocks of nature (the universe) and can be manifested at all fractal levels, independent of how huge or tiny they are as events compared to the whole. This means that the event of “metamorphous top-down” evolution is only a little part of the whole that includes everything from the smallest parts of an atom to the universe itself. But as an isolated event, the evolutionary shift draws our attention because it is a main function of nature, which through evolution has led to the development of us as human beings. This is one of the main reasons why we have drawn our attention specifically to this particular event in this paper.

However, we think the shift in evolution is governed by the superorbital that is formed by gathering all superorbitals from all parts involved in the evolutionary shift (compared to the absolute universe) forming a gathered local “universal” superorbital. This very powerful, local superorbital governs the shift in evolution, from the evolutionary natural selection to the “metamorphous top-down” evolution, through a huge series of biochemical and genetic processes (listed above) that are under tight control from the superorbital (read consciousness), through quantum mechanical activity. All this activity, through “metamorphous top-down” evolution, has led to the development of the human being and its capability for gaining spontaneous healing and self-recovery through adult human metamorphosis[14]. We do not think this is the end of evolution, but through adult human metamorphosis in interaction with evolutionary natural selection, nature has the capability to drive the evolution of human beings to even higher evolutionary goals. By such evolutionary activity, evolution pushes the formation of new peculiar traits necessary for survival for the single human being. Human evolution is still going on, and the most constructive perspective must be that we are on our way to our evolutionary destiny as conscious, healthy, and happy people fully able to use all talents and create optimal value to our world.

## CONCLUSION

It is unlikely that the morphological information of the organism is situated in the DNA base sequences because of the C-value paradox and because the normal self-organization of proteins is unable to explain the general morphology, and because form apparently can evolve without corresponding molecular evolution. According to our hypothesis, the fine structure of energy has an inherent ability to drive the self-organization of all evolutionary levels, from the smallest units to prokaryotes. We believe that there is a creative principle in nature where evolutionary selection works. We believe it makes perfect sense if the fine structure of energy works in a creative way and organizes evolution. The evolution from unicellular to multicellular organisms has also become explainable through our hypothesis, since the creative principle works through creation of still higher organization levels.

We think the “metamorphous top-down” evolution evolved from nature to satisfy its own needs for the creation of huge biological diversity, for its survival as a huge symbiotic ecosystem in the biosphere of the earth. We also think that the “metamorphous top-down” evolution evolved as a result of the single biological individual’s need for fitness and survival in a specific habitat or microhabitat. We think this shift in evolution is guided from the superorbital gathered by the superorbitals from all parts involved in the evolutionary event. We guess that the superorbital governs all genetic and biochemical processes involved in the whole series of events involved in “metamorphous top-down” evolution. Through this we think that nature, by use of its consciousness, has initiated the creation of the great diversity developed through evolution and living on earth today. Furthermore, we think that the gathered superorbital (consciousness) that governs the evolutionary shift is only a local part of the huge superorbital (the main consciousness) of the universe.

We think that “metamorphous top-down” evolution is driven by the consciousness in all organisms from bacteria to humans, but the human consciousness has developed from the “metamorphous top-down” evolution and has given the human being the capability to spontaneously self-heal from mental and physical diseases through a process we call “adult human metamorphosis”. This process is a sophisticated kind of “metamorphous top-down” evolution that makes the human capable of, in interaction with nature, an even higher level of evolution.

## ACKNOWLEDGMENTS

These studies were supported by grants from IMK Almene Fond. Our research in quality of life has been approved by the Copenhagen Scientific Ethical Committee under number (KF)V.100.2123/91.
